# A New and Automated Method for Improving Georeferencing in Nighttime Thermal ECOSTRESS Imagery

**DOI:** 10.3390/s23115079

**Published:** 2023-05-25

**Authors:** Agnieszka Soszynska, Harald van der Werff, Jan Hieronymus, Christoph Hecker

**Affiliations:** 1 Department of Applied Earth Sciences, Faculty ITC, University of Twente, Hallenweg 8, 7522 NH Enschede, The Netherlands; h.m.a.vanderwerff@utwente.nl (H.v.d.W.); c.a.hecker@utwente.nl (C.H.); 2 Department of Computer Science, Humboldt-Universität zu Berlin, 12489 Berlin, Germany

**Keywords:** remote sensing, thermal remote sensing, automated georeferencing, thermal infrared, nighttime imagery, Sentinel-2, ECOSTRESS

## Abstract

Georeferencing accuracy plays a crucial role in providing high-quality ready-to-use remote sensing data. The georeferencing of nighttime thermal satellite imagery conducted by matching to a basemap is challenging due to the complexity of thermal radiation patterns in the diurnal cycle and the coarse resolution of thermal sensors in comparison to sensors used for imaging in the visual spectral range (which is typically used for creating basemaps). The presented paper introduces a novel approach for the improvement of the georeferencing of nighttime thermal ECOSTRESS imagery: an up-to-date reference is created for each to-be-georeferenced image, derived from land cover classification products. In the proposed method, edges of water bodies are used as matching objects, since water bodies exhibit a relatively high contrast with adjacent areas in nighttime thermal infrared imagery. The method was tested on imagery of the East African Rift and validated using manually set ground control check points. The results show that the proposed method improves the existing georeferencing of the tested ECOSTRESS images by 12.0 pixels on average. The strongest source of uncertainty for the proposed method is the accuracy of cloud masks because cloud edges can be mistaken for water body edges and included in fitting transformation parameters. The georeferencing improvement method is based on the physical properties of radiation for land masses and water bodies, which makes it potentially globally applicable, and is feasible to use with nighttime thermal infrared data from different sensors.

## 1. Introduction

Georeferencing accuracy plays a crucial role in providing high-quality ready-to-use remote sensing data. Satellite sensors equipped with star trackers, in addition to GPS, allow a determination of the location and pointing direction, which yields the base for the accurate georeferencing of a remotely sensed image [1]. However, star trackers may malfunction, can be blinded by the sun, or might simply be lacking. Such is the case in the nighttime thermal imagery of the ECOsystem Spaceborne Thermal Radiometer Experiment on the International Space Station (ECOSTRESS). The sensor is mounted on the International Space Station (ISS) and is not equipped with its own star tracker. ECOSTRESS acquires imagery in the thermal infrared (TIR) wavelength range with a spatial resolution of 70 m. If its georeferencing were solely based on the position and attitude of the ISS, errors of approximately 2200 m would appear (which translates to approximately 31 pixels) [2]. Therefore, the standard processing of ECOSTRESS imagery involves image matching to the Landsat Orthobase and is adapted to include TIR imagery [2]. The Landsat Orthobase was created by NASA for internal use and is not publicly available. The product is described at https://landsat.gsfc.nasa.gov/article/nasas-geocover-2000-landsat-product-promotes-geo-commerce-globally (accessed on 6 December 2022). The matching included in ECOSTRESS standard processing is based on 2-D fast Fourier transforms that create tie points between an image and the Landsat Orthobase [3]. The method works well with daytime imagery [2], especially in areas where anthropogenic structures are numerous. However, approximately 62% of all ECOSTRESS scenes do not have successful matching correction [4]. For such cases, the available ISS positioning information is used, and errors of 2.5 km to 7.5 km from the true geographic location can be expected [4].

### 1.1. Georeferencing of TIR Imagery

The accuracy of image matching depends on finding a sufficient number of accurate points that tie the to-be-georeferenced image to the reference [1,5]. Many techniques for the automated finding of such tie points are successfully used for images acquired in the visible (VIS), near-infrared (NIR), and short-wave infrared (SWIR) wavelength ranges (e.g., [5,6,7]). The georeferencing of daytime thermal infrared imagery (TIR) is typically carried out by matching it to VIS imagery acquired simultaneously (e.g., [8,9,10]). These methods fall short in the case of nighttime thermal imagery, and georeferencing errors have been noted for different sensors, such as ASTER [11,12,13] and ECOSTRESS [14]; the authors typically note difficulties in finding ground control points [12]. No specialised methods for georeferencing this kind of imagery can be found in the literature (as of 2023), and researchers using TIR imagery state that the georeferencing of TIR data needs development [15]. Some opt not to georeference TIR imagery at all [15,16] or decide to conduct manual correction [14,17].

ECOSTRESS, as well as other TIR sensors currently operating in space, has a coarser spatial resolution than visible imagery that could be used as a reference [18]. Objects that are typically used as tie points for image matching to a basemap, such as road crossings and the edges of anthropogenic structures [19], are often not resolved in TIR imagery. This is challenging not only for automated procedures but also sometimes even for the manual setting of tie points, which is often performed given the lack of alternatives [14,17]. Additionally, the spatial patterns of surfaces in nighttime thermal infrared can be vastly different from the patterns in the visible (VIS) and short-wave infrared (SWIR) wavelengths or even lack a distinct edge or a common texture [20]. Thus, using a basemap created from VIS-SWIR imagery for the georeferencing of thermal imagery tends to fall short with nighttime imagery.

At the same time, creating a reference basemap based on TIR imagery is challenging due to dynamic changes in the temperature of surfaces during diurnal cycles. The heating of surfaces depends predominantly on solar illumination and weather conditions preceding the time of acquisition. In the case of nighttime TIR imagery, the spatial patterns visible in the image also depend strongly on physical properties that determine the pace of the cooling of surfaces. The pace of cooling down, also known as heat decay, is dependent on heat capacity and emissivity, and consequently, it varies for different materials [20]. Generally, the later in the night, the more time a surface has to cool down. However, cooling happens at different rates for different materials, meaning that surfaces that are warmer than their surroundings after sunset can become colder than their surroundings before dawn. Therefore, each TIR basemap contains signatures for a specific time of day, which will usually not resemble images from other acquisition times. ECOSTRESS acquires imagery at different times of day and night due to the precessing orbit of the ISS, so different spatial patterns can be observed in different images, which makes finding tie points particularly challenging.

### 1.2. Proposed Solution

Our research shows that in areas where infrastructure is scarce (such as the region of the East African Rift), the georeferencing of the tested nighttime images of ECOSTRESS is not particularly accurate, and a mean absolute error of 14.9 pixels is observed. Finding an unambiguous point in natural structures is a challenge for automated tie point finding. A possible solution could be to match the contours of bigger objects using a large number of pixels at a time, thereby increasing the accuracy and reliability of the matched tie points. There is a land cover class that is easy to distinguish in nighttime thermal imagery: water bodies. Water bodies, in contrast to rocks and soil, maintain a relatively stable temperature over the course of a day due to their high heat capacity [21] and constant movement of particles, resulting in high thermal inertia [20]. Land surfaces, on the other hand, tend to have larger diurnal fluctuations in thermal radiation: they are typically warmer than water bodies during the day and colder throughout the night [20]. In consequence, water bodies maintain a high contrast with their surroundings throughout the night, because the moments when their temperature is equal to that of land masses are typically during daylight. This high contrast enables the automatic detection of water bodies in a thermal image, which is favourable for contour matching.

While water bodies usually feature sharp borders, which can be easily detected, the shapes of these borders can vary over time [22]. Due to this dynamic nature, using static spatial data of water bodies is not reliable for purposes such as matching [23]. This applies not only to vector sources but also to raster sources, such as the ASTER Global Water Bodies Database [24], as long as they are not updated regularly. In order to use water bodies as tie points for georeferencing, it is necessary to have an up-to-date reference for each to-be-georeferenced image. Such reference data should be acquired within a short time window (e.g., ±2 weeks) around the acquisition of the target image to maintain the highest possible similarity in the water body extent.

It was stated before that using VIS-SWIR imagery as a reference basemap for TIR imagery does not yield accurate georeferencing results. However, a water body mask derived from such imagery can serve as such. An example of a sensor capable of providing such reference data is Sentinel-2 MSI.

### 1.3. Study Area

The proposed method was tested on imagery of a study area in the East African Rift, located between 3${}^{\circ }$ N 32${}^{\circ }$ E and 5${}^{\circ }$ S 39${}^{\circ }$ E. This area is an example of a region where georeferencing is challenging, both for automated methods and for the manual setting of tie points, because human-made infrastructure is not very visible in the imagery. At the same time, the area contains multiple water bodies of various sizes. The study area is characterised by a mixture of savannah vegetation and bare land. The vegetation percentage varies strongly with time, depending on rainfall occurrence, which typically happens in two rainy seasons.

## 2. Materials and Methods

The descriptions in this section depict the general idea, whereas details, including parameter values and their descriptions, can be found in Appendix B and Appendix C. The scripts used in processing are published on GitHub (the address is provided in the *Data Availability Statement* at the end of the manuscript).

The to-be-improved ECOSTRESS images are further referred to as “target images”, and basemap products used for deriving the correct geolocation are further referred to as “reference images”. The term “matching” is understood as finding the highest overlap between water body edges in a reference basemap and a target image. The term “tie points” is defined as ground control points derived from matched water body edges, from which a final subset is used to fit transformation parameters. A tie point consists of the image coordinates of the water body location in the target image and in its respective reference image. The accuracy of the proposed method was tested using “check points”, which are manually set tie points between the target and reference images.

**Figure 1:**
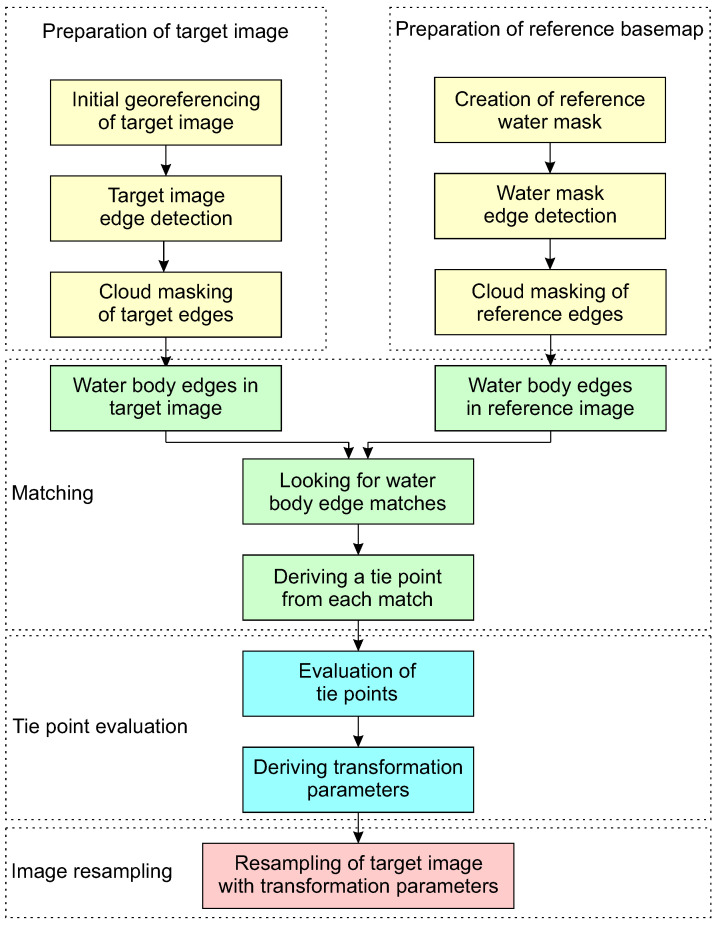
Processing steps of the georeferencing improvement method. Colours symbolise different sections described further in text: yellow for data preparation (Section 2.1), green for matching (Section 2.2), blue for tie point evaluation (Section 2.3), and pink for image resampling (Section 2.4) (creation of final image with improved georeferencing).

The scheme presented in Figure 1 shows the main processing steps in the proposed method. To assess the accuracy of the method, validation was conducted using the manual placement of check points, which is described in Section 2.5.

### 2.1. Data Preparation

#### 2.1.1. Preparation of the Target Images

A set of 23 ECOSTRESS level 2 LSTE (land surface temperature and emissivity) nighttime datasets was used from the 6th processing version with varying cloud cover (the processing version Build 6 is encoded in the file name; see https://lpdaac.usgs.gov/data/get-started-data/collection-overview/missions/ecostress-overview/ (accessed on 6 December 2022), for details on file naming convention). Build 7 was released in autumn 2022, and the reprocessing of the archive will be conducted, with an anticipated finish date in September 2023; however, no significant changes were made to the georeferencing method. The timeframe of the study was limited to the period between the launch in mid-2018 and April 2022. As a selection criterion, only those nighttime ECOSTRESS images that had at least two water bodies visible were used.

To prepare the target layer for processing, the following datasets are required: the image data, cloud mask, and geolocation file. ECOSTRESS LSTE files include temperature image data (land surface temperature, LST layer) and a quality flag layer, from which a cloud mask can be produced. The geolocation file, L1B_GEO, which contains geo-coordinate annotations for each pixel, needs to be downloaded separately. The exact names of the LST datasets we used in this study are provided in Appendix A. The acquisition time and bounding box coordinates of the target data, which are required for the creation of the reference layer, can be found in the metadata.

##### Initial Georeferencing of Target Image Files

The first step in the proposed method is to automatically georeference the target images based on the geolocation information supplied by the standard ECOSTRESS processing chain. The process is initiated by creating a fixed 70 m grid, with the extent derived from the geographic coordinates of the corner pixels. The number of rows and columns of the grid is dependent on the spatial resolution of the scene. The target image is then resampled to the grid using the nearest-neighbour interpolation.

##### Target Image Edge Detection

Next, an edge detection algorithm needs to be applied to the target image. To reduce the influence of radiometric errors and very cold clouds, normalisation is conducted, and the minimum and maximum are set to the 1st and 99th percentiles. In the processing, the Canny edge operator from the OpenCV library [25] is used to obtain a binary target edge image from the ECOSTRESS LST image. By adjusting the Canny edge operator parameters (upper and lower threshold values in hysteresis thresholding), more or fewer edges will be derived. These parameters are calculated for each image separately based on the median of the image LST and the adjustment of the parameter “threshold_sigma” (see Table A2 for details).

After edge detection, the derived edges are subsequently dilated to match the water body edges to the reference more reliably. If the water body extent in the reference and target images differs by even 1 pixel, the matching process will fail more frequently. Even such small a difference in the water extent will cause only a part of a water body edge to be matched, which will subsequently not fulfil the validity criteria (which are described later). Such differences can appear when vegetation is present on the edges of a water body, for example, reeds. In such a case, this area in the reference layer can be classified as vegetation, whereas the thermal signal in the target image can be still high enough to be included in the water body edges.

##### Cloud Masking of Target Edges

As a final step, cloud masks from both the target and reference are applied to the target edge image, as well as a bounding box mask, which should cover the edges of the image. As the reference and target images are not acquired at the same time, different areas will be covered by clouds. By using both target and reference cloud masks on both reference and target edges, the uncertainty caused by the inaccuracy in the extent of water bodies is reduced. The cloud masks and bounding box mask edges are dilated to ensure that only the edges of land cover surfaces are visible in the target edge image.

In ECOSTRESS processing, an additional statistics-based cloud mask is used, which was added because the original cloud masks provided by quality flags can contain errors. The statistics-based cloud mask is created by thresholding, for which the value is derived by fitting a Gaussian function to the LST histogram (see Table A1 for details). All pixels in which the LST value is below 1.5σ of the fitted Gaussian distribution are masked.

The principle of cloud masking was changed for Build 7 of ECOSTRESS standard processing, and much higher masking accuracy was obtained [26,27]. Most likely, the additional cloud masking can be turned off for imagery processed with Build 7.

#### 2.1.2. Preparation of the Reference Layer

For creating a reference basemap, data from Sentinel-2 MSI were chosen. With two sensors (S2A and S2B) currently operational, the Sentinel-2 mission has a repeat overpass of 5 days, which provides a high probability of obtaining cloud-free data in a given area [28]. The Sentinel-2 MSI instruments have 13 multispectral bands covering VIS, NIR, and SWIR wavelengths with a 290 km swath width [29]. The absolute geolocation error of Sentinel-2 imagery is reported to be a maximum of 7.1 m for S2A and 4.6 m for S2B (at 95% confidence) [30]. The ground sampling distance (GSD) of Sentinel-2 MSI (10 m, 20 m, and 60 m for different spectral bands) is finer than that of currently available TIR sensors (the highest spatial resolution of 60 m is Landsat-7), which is advantageous for creating a reference image. The short revisit time (allowing for avoiding cloud cover), worldwide coverage, high spatial resolution, and good georeferencing accuracy make this sensor an optimal choice for creating an up-to-date reference for nighttime thermal images.

The preparation of the reference layer starts with creating a mosaic of water masks from imagery acquired in a given time window. To prepare the reference, the coordinates of the target image bounding box and a time window around the acquisition of the target image need to be defined. The larger the time window, the higher the probability of finding cloud-free imagery for the reference, but also the higher the probability of a land cover change. A time window covering a month when the acquisition of the target image was conducted was chosen. This range is suitable for the analysed study area, since water body changes happen in the range of months and years rather than days.

##### Creation of the Reference Water Mask

Google Earth Engine (GEE) [31] is used to collect and process Sentinel-2 MSI data acquired on given dates. The “Scene Classification Layer” (SCL) that comes with the level-2 product is a land cover map with 20 m spatial resolution [32,33]. For the purpose of generating a monthly reference image of water bodies, the SCL is aggregated per calendar month, resulting in 12 such products for each year. Additionally, shadow, snow, and cloud classes are used to mask the missing information in the reference layer when water would be invisible. The GEE Javascript code, including details, for preparing these “water masks” is provided in Appendix B.

For further processing, the water mask from the acquisition month of a respective target image is chosen. The chosen water mask is reprojected from the original spatial resolution of 20 m to match the projection of ECOSTRESS data at 70 m.

##### Water Mask Edge Detection

To create the actual reference basemap, an edge operator is applied to the reprojected water mask so that water body edges are derived. The water body edges in the reference are dilated in the same manner as in the target edge image to enhance the stability of matching. In the processing, the Canny edge operator is used to derive the edges of reference water bodies.

##### Cloud Masking of Reference Edges

As a final step, the reference basemap is cloud-masked using the reference cloud mask and both cloud masks from the target image.

### 2.2. Matching Edges of Water Bodies between Target and Reference Images

During this processing step, a match for each water body in the reference image is sought in the target image. If a match is found, a tie point is derived, which is later tested for accuracy and reliability.

In the processing, a matching procedure based on a “brute force” principle is applied to compare the overlap of the edges of water bodies in the target and reference images within a search window. This process starts with identifying the edges of each water body in the reference layer. For each water body, a search window in the target edge image extending from the reference water body location is extracted; it contains the extent of the whole water body, as well as the maximum presumed shift. The size of the search window should be defined as slightly larger than the maximum expected georeferencing error. In the datasets used, errors of up to 65 pixels can be observed, so the searched window was defined as ±75 pixels from the correct position (derived from the reference). However, it is important to note that this parameter has the highest influence on processing time.

**Figure 2:**
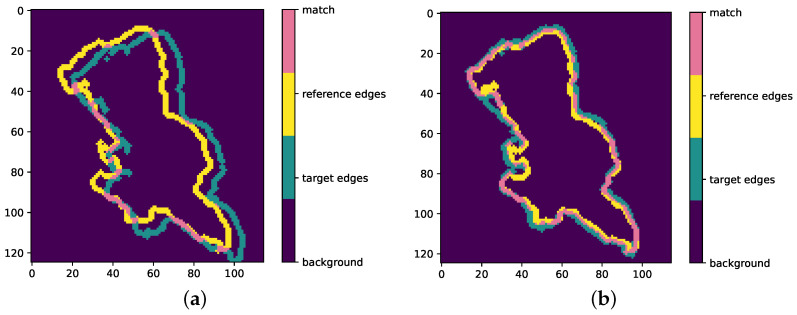
Illustration of the matching procedure. Overlap between the reference and target edges is tested for each position in the search window. If the overlap is not optimal (**a**), no tie point will be derived. The position with the maximum overlap (**b**) yields a tie point.

Next, the extraction from the target edge image (of the size of the water body in the reference image) is compared to the reference (Figure 2). The extracted image fragment is iteratively shifted over the search window, one pixel at a time in x- and y-directions, and a comparison of the overlap between the reference and target is repeated for each position. The overlap in each position is evaluated by adding the binary edges in the reference and target fragments and is expressed by a histogram of image values: 0 (background), 1 (not matching edges), and 2 (matching edges). The optimal position is subsequently given by the highest number of overlapping edges and saved as a *tie point*.

### 2.3. Tie Point Evaluation

After iterating through all the labelled water bodies in the reference image, a set of tie points is obtained. Some of these tie points could be incorrect (for instance, due to edges of cloud remnants and features of the Earth’s surface), and these need to be identified and removed so that only correct tie points remain. Thus, several criteria for evaluating the tie points were implemented to increase the probability of keeping only the correct tie points. In order to derive three transformation parameters (x-offset, y-offset, and rotation), at least two valid tie points need to be found. It is also possible to include more transformation parameters; however, a larger number of reliable tie points is needed in such cases.

Several selection criteria were defined in the processing to evaluate tie points:

- Number of edge pixels in the considered water body;
- Number of matching (overlapping) edge pixels;
- Number of matching pixels with respect to all edge pixels of the water body;
- Distribution of the matching pixels in the water body.

The example presented in Figure 2 shows both matching and non-matching pixels in a water body. A tie point can be regarded as reliable when a water body is large, when there are many matching pixels (absolute value as well as relative to all edge pixels), and when the matching pixels are equally distributed over different parts of the water body.

An additional evaluation step is performed by deriving transformation parameters from a subset of tie points and subsequently removing tie points that produce transformation parameters that are inconsistent with the majority. Euclidean distances between tie point image coordinates in the reference image and transformed coordinates are calculated as residuals, and points exceeding a given threshold are treated as outliers. A threshold value of 3 pixels and the 80th percentile frequency of high residual error in combinations of tie points were used to reject outlying tie points. After removing incorrect tie points, the final transformation parameters are determined if two or more valid tie points remain.

### 2.4. Image Resampling

The last step is to transform the original target image with the newly derived transformation parameters. Nearest-neighbour resampling is applied to ECOSTRESS images, and, additionally, the quality flag annotation files are transformed as well.

After resampling, transformation metadata for each image are generated, which includes Euclidean distances between image coordinates in the transformed target image and coordinates in the reference layer for each tie point. These distances provide an error for each tie point and further mean an error for each target image. Moreover, they serve as information on the residual error and an accuracy measure of the transformation, as well as the reliability of the transformation given by the number of tie points used.

### 2.5. Validation

To test the accuracy of the proposed method, the target images transformed into reference basemaps were compared by manually setting ground control points (check points) in both images. A set of check points evenly distributed over each entire image was defined so that errors related to rotation were also retrieved. The check points were set in places that could be unambiguously identified in the target images, such as at corners in water body edges, and a large spread of check points throughout the image was sought. However, the number of check points per image is strongly dependent on cloud cover, image quality, and the visibility of unambiguous features and consequently varies between images.

For each image, Euclidean distances for each check point were calculated, and additional statistical parameters of all the check points were saved as further transformation metadata. The same procedure was performed for original images provided by standard processing; thus, a comparison between the accuracy obtained with the proposed method and the accuracy from the original processing could be made.

## 3. Results

**Figure 3:**
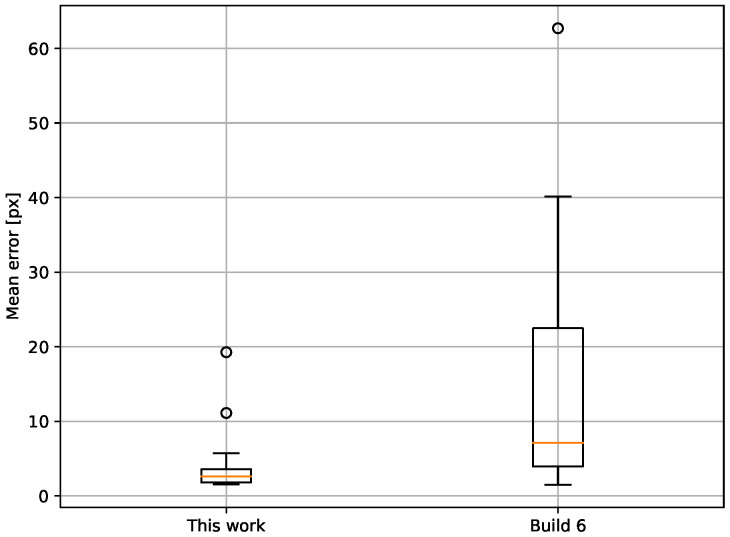
The mean georeferencing error in images obtained with the proposed method (“This work”) and with the standard ECOSTRESS processing (“Build 6”).

**Figure 4:**
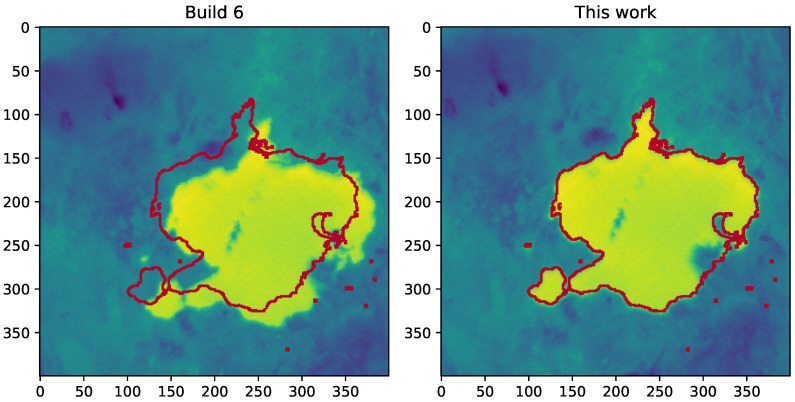
An example of georeferencing accuracy for image 20210923T000600 after standard processing (“Build 6”) and the proposed method (“This work”), expressed as an overlap between reference edges of a water body (marked red) and the ECOSTRESS image.

The boxplots in Figure 3 show the difference between the georeferencing provided by the data supplier and the results of the proposed method for the tested set of images. Out of the 23 images used in this test, 22 could be processed. The remaining dataset could not be processed, because the ECOSTRESS cloud mask covered all water bodies in the area (although the water bodies were visible in the LSTE image). An example of georeferencing improvement between the original processing and the proposed method is illustrated in Figure 4. The reference edges of Lake Naivasha (marked red) do not overlay the water body in the ECOSTRESS image after the original processing (“Build 6”) but do after the processing chain presented in this paper (“This work”).

The fitted transformation parameters for each dataset can be seen in Table 1. Most of the transformations are x- and y-offsets, while the rotation fitted for most images does not exceed 0.1°.

Table 2 shows the number of tie points used for fitting the transformation parameters and the mean Euclidean distances between the reference and transformed tie points. Generally, the higher the number of tie points used, the more reliable the transformation parameters. However, it is important to remember that even a single incorrect tie point can strongly influence the whole transformation. Therefore, if all the distance values per image are similarly low, the transformation is likely to be accurate, whereas outliers suggest an error. The mean distances per image presented in Table 2 range between 0.1 and 2.1  pixels; the overall average error is 1.3  pixels.

The detailed accuracy results, including Euclidean distances for each check point, are presented in Table 3 and Table 4 for images processed with the proposed method and images processed with the standard processing of ECOSTRESS (Build 6), respectively. The number of check points for each dataset depends on the cloud cover and image quality.

**Figure 5:**
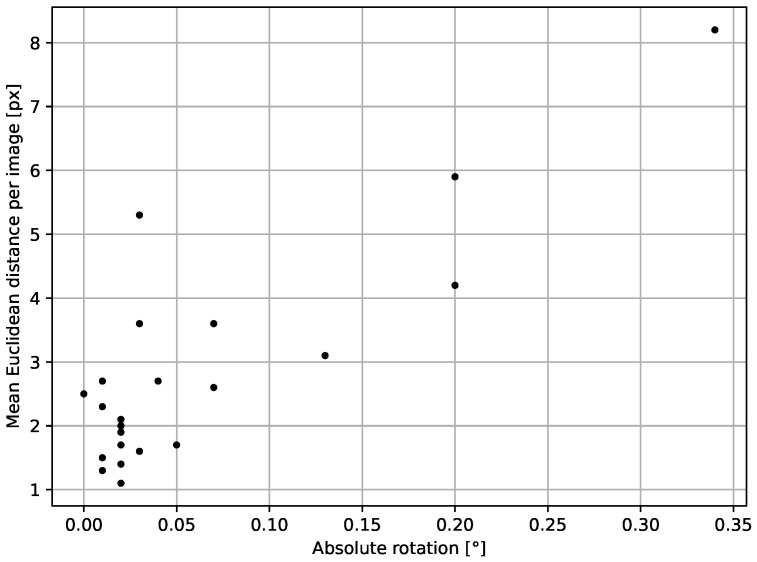
Relation between mean absolute error and rotation parameter. R^2^ for this relation is 0.71.

The accuracy results (presented in Table 3) show a considerable spread of Euclidean distances. This seems to happen when the tie points used to define the transformation matrix are located in only one part of the image: errors appear in areas distant from the tie points, especially when an image is rotated (Figure 5). It seems that higher rotation values cause an overcorrection. The largest error appears for image 20210717T025340, which was processed with only two tie points.

**Figure 6:**
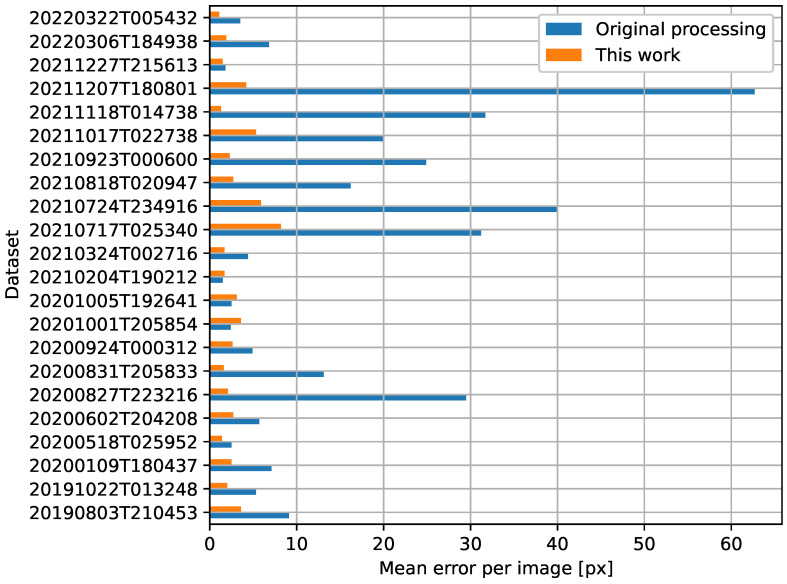
Error in the original processing (“Build 6”) and in the processing with the proposed method (“This work”), expressed as the mean Euclidean distance of the check points for each image.

A strong improvement in georeferencing with the proposed method is supported by Figure 6, which illustrates the difference between the mean error (Euclidean distance) in the original processing (Build 6) and that obtained in this work. In 19 out of 22 images, the values from the proposed method (marked orange) are lower than the values from the original processing (marked blue): on average, 12.0  pixels. In three cases, the error slightly increased in comparison to the standard processing; however, the overall trend shows improvement.

## 4. Discussion

High georeferencing accuracy is a crucial requirement for any remote sensing analysis. A new approach to georeferencing improvement is proposed, specifically designed for thermal IR nighttime imagery. The proposed method was tested on a set of nighttime ECOSTRESS LST images of the East African Rift, and a georeferencing improvement of 12.0  pixels on average was noted, while an average accuracy of ±2.9 pixels was achieved. The improvement in accuracy is supported by Figure 6, which presents a comparison between the mean error per image from the original processing and from the proposed method. There are only two cases in which the proposed method led to larger errors than in the original processing, but the absolute values of these errors are rather small (Figure 6). On average, the georeferencing error decreased by 12.0  pixels, from 14.9 pixels to 2.9 pixels.

### 4.1. Analysis of the Method

The proposed method assumes that water body edges can be easily identified in TIR nighttime imagery and that the same edges can be identified in the reference. The first assumption is usually true due to the heat capacity and thermal inertia differences between land and water; however, in cases where the LST difference between land and the water body is small, adjustments to edge detection should be made. The Canny edge detection algorithm allows the adaptation of thresholds for the hysteresis procedure (in this implementation, it was adapted by using the parameter “threshold_sigma”; see Appendix C Table A2 for details). As for the similarity with the reference, the water bodies in the land cover classification are easy to identify due to their strong spectral differences from land masses and vegetation. The LST of water bodies at night is higher than that of the land masses, and therefore, the edges in the reference and target images are similar enough for matching purposes (the few exceptions to this are discussed below).

The proposed method can be applied globally, provided that some water bodies are present and visible in the image. Since ECOSTRESS scenes have a 384 km swath width, chances are that at least some water bodies are present in an image, although areas with snow cover probably need to be excluded.

#### 4.1.1. Implementation

As there is no dedicated method for improving the georeferencing of nighttime TIR imagery, the results obtained were compared to the standard processing of ECOSTRESS. The tie point residuals (presented in Table 2) complement the information provided by manual checkpoint setting; these values have an average accuracy of ±1.3  pixels. Since the residuals are provided as metadata for each processed image, they can be used by all users for the preliminary assessment of the transformation accuracy and reliability. If Euclidean distances are similarly low for all tie points, georeferencing is likely to be accurate, whereas singular larger distances suggest a larger error, e.g., due to rotation error.

Limitations of the validation procedure need to be considered in the analysis. The distribution of check points is supposed to be as homogeneous as possible, and some check points should be placed at the edges of each image. However, cloud cover, radiometric artefacts, and the lack of unambiguous features to serve as check points influenced the checkpoint distribution and, therefore, may have introduced a bias into the validation dataset.

It is important to note that the proposed method considers the most up-to-date reference image but does not accommodate rapid events (e.g., weather or anthropogenic) that change the contours of water bodies. In the processing, a temporal resolution of one month was used, which is sufficient for the analysed study area, where land cover changes happen over the course of seasons. However, for other study areas, the time window may need to be adjusted. For instance, if there is a dynamic change in the water level, a temporal resolution of one month may not be enough. At the same time, it is important to remember that decreasing the time window can come at the cost of data gaps, because fewer datasets will be available to create a cloud-free mosaic.

A special case of dynamic change to consider is a seashore area with high tides. If enough inland water bodies are present, sea areas can be masked and disregarded in the matching. In principle, a reference with the same tide level can be used; however, finding a cloud-free reference with the same tide level may be challenging.

#### 4.1.2. Data Quality

The proposed method depends on the visibility of matching objects on the Earth’s surface; therefore, the largest weakness in the processing comes from imprecise cloud masking in both target and reference data. If clouds are not masked properly, their edges will be used in feature matching, thereby introducing errors. The addition of a statistics-based cloud mask based on the LST histogram was introduced to reduce errors from cloud edges. Several tie point evaluation steps were implemented to remove invalid tie points based on the matching of cloud edges, but some erroneous tie points may nevertheless remain.

Additionally, some errors can appear due to georeferencing errors of cloud masks, because cloud masks of both the target and reference are used on both datasets. Thus, if a cloud covers a water body in the target image, but the georeferencing of these masks has an offset, the matching may be faulty. Such errors are possible despite the evaluation of tie points; however, no such cases were observed in the images processed.

Errors can also appear due to other reasons. The reference images, which are binary masks, were resampled from their original spatial resolution of 20 m to match the spatial resolution of ECOSTRESS imagery at 70 m, which can potentially introduce a shift in the geolocation of water body edges. Additionally, the reference imagery was acquired at different wavelengths from those used to obtain the target images. Differences may especially appear in areas where land cover is more complex than the classes provided in the SCL, such as vegetation floating on water. In the SCL, such areas will be classified as vegetation, but in ECOSTRESS images, they are seen as warmer than land surfaces, and in processing, they are treated as water. Additionally, assumptions were made to create the classification layer, and these assumptions may lead to differences in the delimitation of a water body.

Lastly, misclassification errors in the SCL additionally add to the overall error per image. The risk of maintaining an error in the reference image, however, is minimised due to the fact that for each month of acquisitions, a separate reference layer is created.

#### 4.1.3. Finding and Evaluating Tie Points

The proposed matching algorithm uses a brute force principle by comparing image fragments and shifting these fragments pixel by pixel. This allows the x-offset, y-offset, and rotation to be derived for each image. In the manual assessment of ECOSTRESS imagery, no images were encountered for which a transformation including scaling or shearing would be necessary, so we decided to opt for fewer transformation parameters and a lower minimum number of tie points for the improvement process to take place. However, since ECOSTRESS has a swath width of approximately 400 km, errors can appear in higher off-nadir angles, especially in the most extreme pixels. The implementation of the method, however, allows for fitting scaling and shearing. The number of parameters that can be fitted depends on the number of valid tie points found, so the adaption of the parameter “minimum_num_tp” would be required to fit additional parameters.

The proposed method does not consider rotation for each water body separately, and the geometric resolution of a tie point is limited to a pixel. The results presented in Figure 5 suggest an overcorrection in cases where rotation exceeds 0.2${}^{\circ }$. However, due to different manoeuvres taking place on the ISS, rotation in the imagery can appear, so disregarding this parameter does not seem to be a correct solution.

In this implementation, two parameters limiting the water body choice have been defined: the minimum size of the water body and the minimum number of detected edge pixels. In the study area, numerous small, round water bodies can be found, and their similarity may lead to difficulties in fitting transformation parameters. We decided to reduce the number of matching objects to large water bodies, which typically have more complex shapes, in order to avoid introducing mismatches. Potentially, algorithms, such as Iterative Closest Point, could enhance the precision of matching. In areas with multiple small water bodies, constellations thereof could be used for matching, with a similar principle to that used in star trackers.

The reliability of the proposed georeferencing method strongly depends on the number of matches found during the process. It appears that images with the highest error values were processed with only a few tie points (Table 2 and Table 3). Generally, the more tie points used for fitting the transformation parameters, the more reliable the method becomes. However, keeping even a single wrong tie point means that all transformation parameters for the complete image will be wrong. Therefore, we decided to opt for using fewer tie points, but with higher restrictions on accuracy. This principle works well as long as no scaling or shearing is present. Nevertheless, errors can also appear if tie points in a group are located within small distances to each other and one tie point is located in the distant part of the image, because the matching error for this single point is much harder to identify. Such a situation may lead to the overcorrection of rotation, which is possibly the case for 20210717T025340, 20210724T234916, 20211207T180801, and 20201005T192641. This is visible when analysing the standard deviation of errors (Table 3). For instance, in image 20201005T192641, one out of three tie points (Table 2) is an invalid match but was not picked out in the tie point evaluation step. This wrong tie point influenced the whole transformation matrix, resulting in an average checkpoint error of 4.3 pixels for this image (Table 3).

The parameters in the evaluation criteria were empirically derived and possibly need adaption for other study areas. There is a trade-off between the strictness of the evaluation and the number of images that will not be processed at all due to insufficient remaining tie points. The user needs to decide upon the rigour of the tie point evaluation, considering the application.

### 4.2. Future Directions

The proposed method can be optimised in the future, e.g., by using algorithms such as Iterative Closest Point, including the rotation of each individual water body, accounting for sub-pixel offsets, and using constellations of small water bodies for matching.

The method can potentially be applied to imagery from different sensors, because water bodies maintain a high contrast with the surrounding land masses throughout the night. As new operational thermal sensors appear (e.g., TRISHNA, Surface Biology and Geology and Land Surface Temperature Monitoring, as well as low-cost commercial constellations), applications using nighttime TIR imagery will gain importance. The ability to accurately georeference imagery in an automated way enables the provision of automated solutions on a global scale, and the proposed method has the potential to support this process.

With the increasing availability of computational power, it is possible to focus on better image-matching approaches compared to object-based matching. The presented research proves that using an up-to-date reference solves the issue of outdated reference basemaps due to dynamically changing land cover. Since the preparation of a large mosaic for referencing only takes a few seconds in a cloud-based environment such as GEE, it is possible to use the most recent images from high-resolution operational satellites with high georeferencing accuracy as a reference, instead of large reference databases such as the Landsat Orthobase. In the processing, the reference masks were downloaded from GEE, and the process ran locally (which takes approximately 25 min per image), but if the approach were reversed and target images were uploaded to a cloud computing platform, the overall processing time might be strongly reduced.

## 5. Conclusions

Standard matching algorithms often fall short in the georeferencing of thermal imagery because of dynamically changing patterns and the spectral autocorrelation of thermal spectral bands. The difference between the proposed method and standard approaches is twofold: (1) an up-to-date reference is created for each image, which accounts for seasonal and long-term changes in land cover classes, and (2) to create a tie point, a large number of pixels, forming the majority of the water body edge, must match with the reference. This allows a more robust tie point identification in an environment where few tie points are found in the standard processing of ECOSTRESS. The achieved average accuracy of 2.9 pixels shows that matching nighttime thermal IR images to an up-to-date land/water mask leads to successful automatic tie point creation between the image and the reference. The results are reliable and robust as long as several water bodies are contained in the scene. In the case of the tested nighttime ECOSTRESS images, the proposed method improved the georeferencing by 12.0  pixels compared to standard processing. With a shift towards cloud computing, it is possible to both create a reference for each image and conduct the entire processing on the fly in the cloud.

## Figures and Tables

**Table 1:** Fitted transformation parameters.

Dataset	Rotation (°)	X-Offset (px)	Y-Offset (px)
20190803T210453	−0.03	−10.2	−1.1
20191022T013248	−0.02	−4.2	4.6
20200109T180437	0.00	−3.5	7.1
20200518T025952	−0.02	0.0	1.1
20200602T204208	−0.04	−10.4	2.3
20200827T223216	0.02	11.6	−27.1
20200831T205833	0.03	−9.7	4.2
20200924T000312	0.07	0.5	−5.8
20201001T205854	−0.07	−6.9	7.8
20201005T192641	0.13	6.6	−11.5
20210204T190212	0.05	2.3	−3.7
20210324T002716	−0.02	−0.5	−2.9
20210717T025340	−0.34	−9.7	49.6
20210724T234916	−0.20	15.0	47.6
20210818T020947	0.01	6.9	−14.9
20210923T000600	0.01	−19.1	−15.1
20211017T022738	0.03	−15.6	7.6
20211118T014738	−0.01	−20.4	−22.9
20211207T180801	−0.20	−43.8	−40.0
20211227T215613	−0.01	−2.2	0.1
20220306T184938	−0.02	−0.8	−4.9
20220322T005432	0.02	−1.2	−3.1

**Table 2:** Euclidean distances between reference and transformed target tie points. The table indicates the number of evaluated tie points per image and their residual errors. Large residuals (e.g., <5.0) may indicate incorrect tie points that require further investigation or the adjustment of parameters.

Image	Mean (px)	Number of Tie Points Used	Tie Points Euclidean Distance (px)							
20190803T210453	1	7	0.6	1.1	0.4	0.8	1.4	1.2	1.2	
20191022T013248	0.4	4	0.3	0.8	0.5	0.2				
20200109T180437	1.8	5	2	0.8	1.8	2.5	2.1			
20200518T025952	1.1	7	1	0.4	2	1.4	0.5	1.3	1	
20200602T204208	0.1	2	0.1	0.1						
20200827T223216	0.9	3	0.4	1.3	0.9					
20200831T205833	1	6	0.8	1.1	1.2	1.9	0.3	0.8		
20200924T000312	1.3	8	1.6	0.9	0.4	1.4	1.6	2	1.1	0.9
20201001T205854	2.1	3	2.4	0.9	3					
20201005T192641	1.9	4	2.1	2.1	1.1	2.1				
20210204T190212	1.6	8	2.6	1.3	0.9	1.1	1.1	1.8	1.2	2.8
20210324T002716	1.4	8	1.3	2.1	0.4	0.3	2.8	1.3	1	1.9
20210717T025340	1.8	2	1.8	1.8						
20210724T234916	0.5	3	0.7	0.6	0.3					
20210818T020947	1.3	6	1.3	0.7	1.7	1.1	2	1.1		
20210923T000600	1.8	6	1.4	0.9	1.9	2.2	1.8	2.4		
20211017T022738	1.1	6	0.9	0.3	1	1.8	0.7	2.1		
20211118T014738	1.3	5	2.3	0.9	1	1.5	0.9			
20211207T180801	1.6	4	1.8	2.2	0.3	2.1				
20211227T215613	1.7	6	0.8	1.6	2.4	2.1	1.5	1.7		
20220306T184938	1.4	5	0.9	1.3	2.5	0.8	1.5			
20220322T005432	1.1	7	1	1.4	1.7	0.8	1.4	0.3	1.3	

**Table 3:** The mean (μ), median (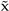), and standard deviation ($\sigma_{x}$) of the error derived from manual checkpoint setting in images transformed with our method. The mean error for all the images is 2.9.

Image	Check Point Euclidean Distance (px)											μ	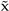	$\sigma_{x}$
20190803T210453	3.5	7.1	0.7	0.6	2.5	0.4	1.0	4.4	8.7	8.2	2.8	3.6	2.8	2.9
20191022T013248	1.3	1.0	1.5	4.2	1.2	1.8	2.6	2.2				2.0	1.6	1.0
20200109T180437	0.4	2.8	2.0	4.6	3.6	2.4	1.6					2.5	2.4	1.3
20200518T025952	0.6	0.7	4.0	0.8	0.9	1.0	1.0	2.6				1.4	0.9	1.1
20200602T204208	3.6	2.2	2.5	1.0	3.0	2.4	4.4	2.4				2.7	2.5	0.9
20200827T223216	1.5	1.6	2.2	1.6	3.2	2.2						2.1	1.9	0.6
20200831T205833	0.7	1.1	0.9	0.9	2.3	1.8	0.2	3.0	3.3	2.0		1.6	1.4	1.0
20200924T000312	0.3	4.4	3.3	4.0	2.6	1.6	3.1	1.9				2.6	2.8	1.3
20201001T205854	7.3	7.0	2.2	1.7	4.3	0.4	4.3	1.3				3.6	3.2	2.4
20201005T192641	6.7	2.1	0.8	5.0	2.3	1.3	3.5					3.1	2.3	2.0
20210204T190212	2.0	1.0	2.7	1.0	1.4	1.3	3.8	0.8				1.7	1.3	1.0
20210324T002716	1.3	2.6	1.1	2.2	1.6	2.3	1.2	0.9	1.6	2.2	2.1	1.7	1.6	0.5
20210717T025340	19.0	2.0	5.1	6.5	3.1	11.1	10.9					8.2	6.5	5.5
20210724T234916	6.4	4.0	0.5	3.3	9.2	7.3	11.0					5.9	6.4	3.4
20210818T020947	5.0	4.7	5.2	0.9	3.0	0.4	2.1	0.5				2.7	2.6	1.9
20210923T000600	2.5	0.8	4.5	1.5	3.7	0.6						2.3	2.0	1.4
20211017T022738	7.6	7.6	1.4	4.2	8.5	3.6	4.2					5.3	4.2	2.5
20211118T014738	1.2	1.6	1.1	1.7	0.7							1.3	1.2	0.4
20211207T180801	2.0	2.5	7.5	2.5	6.3	4.2						4.2	3.4	2.1
20211227T215613	0.8	1.5	2.6	0.8	1.8	1.3						1.5	1.4	0.6
20220306T184938	1.3	0.8	1.5	0.7	1.2	1.5	2.6	3.1	1.4	5.0		1.9	1.4	1.3
20220322T005432	2.5	1.5	0.2	1.3	0.7	1.2	0.2					1.1	1.2	0.7

**Table 4:** The mean (μ), median (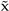), and standard deviation ($\sigma_{x}$) of the error in the original processing (Build 6), derived from manual checkpoint setting. The mean error for all the images is 14.9 px.

Image	Check Points Euclidean Distance (px)							μ	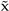	$\sigma_{x}$
20190803T210453	9.0	9.0	9.5	9.7	8.1			9.1	9.0	0.5
20191022T013248	6.7	5.3	5.7	4.9	4.1			5.3	5.3	0.9
20200109T180437	7.9	6.7	8.2	6.2	5.5	8.0		7.1	7.3	1.0
20200518T025952	2.5	2.3	4.7	1.4	1.6			2.5	2.3	1.2
20200602T204208	5.0	3.0	5.5	9.2	8.5	4.6	3.7	5.7	5.0	2.2
20200827T223216	28.9	27.9	29.3	33.0	30.4	27.6		29.5	29.1	1.8
20200831T205833	10.2	13.7	13.4	15.4	12.6	13.4		13.1	13.4	1.6
20200924T000312	4.6	4.6	4.3	4.4	3.8	7.3		4.9	4.5	1.1
20201001T205854	2.0	3.8	1.9	2.4	2.5	1.8		2.4	2.2	0.7
20201005T192641	3.6	1.2	3.4	2.4	2.0			2.5	2.4	0.9
20210204T190212	0.8	1.1	2.2	2.1	0.0	2.6		1.5	1.6	0.9
20210324T002716	5.1	2.8	4.6	6.7	3.0	4.3		4.4	4.4	1.3
20210717T025340	30.7	32.2	31.5	32.1	29.4			31.2	31.5	1.0
20210724T234916	26.4	42.1	44.8	47.2				40.1	43.5	8.1
20210818T020947	17.8	15.6	16.0	17.0	13.9	16.7		16.2	16.4	1.2
20210923T000600	25.4	23.8	24.9	25.6				24.9	25.2	0.7
20211017T022738	16.9	27.0	17.9	19.9	18.5			20.0	18.5	3.6
20211118T014738	31.3	30.9	34.0	30.8				31.7	31.1	1.3
20211207T180801	61.1	65.1	61.2	62.9	63.2			62.7	62.9	1.5
20211227T215613	1.1	2.2	2.6	2.1	2.1	0.7		1.8	2.1	0.7
20220306T184938	8.7	5.9	5.8	7.3	5.8	7.2		6.8	6.6	1.1
20220322T005432	5.2	3.3	3.1	1.7	3.9			3.5	3.3	1.1

## Data Availability

Scripts used for data processing are available at https://github.com/marchewaka/Nighttime_TIR_georeferencing_improvement_ECOSTRESS.

## Abbreviations

    The following abbreviations are used in this manuscript:

ASTER	Advanced Spaceborne Thermal Emission and Reflection Radiometer
ECOSTRESS	Ecosystem Spaceborne Thermal Radiometer Experiment
	on the International Space Station
GEE	Google Earth Engine
GSD	Ground Sampling Distance
ISS	International Space Station
LST	Land Surface Temperature
LSTE	Land Surface Temperature and Emissivity
MSI	MultiSpectral Imager
NIR	Near InfraRed
SCL	Scene Classification Layer
SWIR	Short-wave InfraRed
TIR	Thermal InfraRed
VIS	Visible

## Appendix A. List of Image Identifiers

**File ID**	**Paper ID**
ECOSTRESS_L2_LSTE_06108_017_20190803T210453_0601_02	20190803T210453
ECOSTRESS_L2_LSTE_07336_021_20191022T013248_0601_02	20191022T013248
ECOSTRESS_L2_LSTE_08572_020_20200109T180437_0601_01	20200109T180437
ECOSTRESS_L2_LSTE_10578_018_20200518T025952_0601_01	20200518T025952
ECOSTRESS_L2_LSTE_10822_017_20200602T204208_0601_01	20200602T204208
ECOSTRESS_L2_LSTE_12156_025_20200827T223216_0601_01	20200827T223216
ECOSTRESS_L2_LSTE_12217_021_20200831T205833_0601_01	20200831T205833
ECOSTRESS_L2_LSTE_12576_007_20200924T000312_0601_01	20200924T000312
ECOSTRESS_L2_LSTE_12698_016_20201001T205854_0601_01	20201001T205854
ECOSTRESS_L2_LSTE_12759_016_20201005T192641_0601_01	20201005T192641
ECOSTRESS_L2_LSTE_14650_016_20210204T190212_0601_01	20210204T190212
ECOSTRESS_L2_LSTE_15383_015_20210324T002716_0601_01	20210324T002716
ECOSTRESS_L2_LSTE_16001_021_20210502T203637_0601_01	20210502T203637
ECOSTRESS_L2_LSTE_17172_016_20210717T025340_0601_01	20210717T025340
ECOSTRESS_L2_LSTE_17294_018_20210724T234916_0601_01	20210724T234916
ECOSTRESS_L2_LSTE_17670_030_20210818T020947_0601_01	20210818T020947
ECOSTRESS_L2_LSTE_18228_017_20210923T000600_0601_01	20210923T000600
ECOSTRESS_L2_LSTE_18602_028_20211017T022738_0601_01	20211017T022738
ECOSTRESS_L2_LSTE_19099_015_20211118T014738_0601_01	20211118T014738
ECOSTRESS_L2_LSTE_19404_017_20211207T180801_0601_01	20211207T180801
ECOSTRESS_L2_LSTE_19716_033_20211227T215613_0601_01	20211227T215613
ECOSTRESS_L2_LSTE_20784_022_20220306T184938_0601_01	20220306T184938
ECOSTRESS_L2_LSTE_21021_016_20220322T005432_0601_01	20220322T005432

## Appendix C. Parameters Used in the Georeferencing Improvement Process

**Table A1:** Parameters used for statistics-based cloud masking.

Parameter	Value	Explanation
minimum_LST	250	Minimum expected valid LST value in K (including clouds).
maximum_LST	320	Maximum expected valid LST value in K (including clouds).
cloud_masking_num_sigmas	1.5 ×σ	Multiplier for sigma, giving threshold value for cloud masking.

**Table A2:** Parameters used for calculation of edge image.

Parameter	Value	Explanation
threshold_sigma	0.33	Canny edge parameter calculation. A lower value of sigma indicates a tighter threshold, whereas a larger value gives a wider threshold.
kernel_size_masking	9×9	Kernel size for dilation of cloud masks and bounding box.
kernel_edge_dilation	$\begin{array}{ccc} 0 & 1 & 0\\ 1 & 1 & 1\\ 0 & 1 & 0 \end{array}$	Kernel used for edge dilation.

**Table A3:** Parameters used for matching water body edges.

Parameter	Value	Explanation
minimum_lake_size	50	Minimum size of a water body in pixels to be considered.
test_range_span	75	Half of the search window size.
minimum_edge_size	10	Minimum number of edge pixels for a water body to be considered.
create_control_plots	False	If true, plots for individual matched water bodies will be saved.

**Table A4:** Parameters used for tie point evaluation.

Parameter	Value	Explanation
importance_evaluation_test_1	60	For water bodies of this size, additional importance is given.
importance_evaluation_test_2	200	For water bodies of this size, additional importance is given.
importance_evaluation_test_3	100	For water bodies of this size, distribution test is conducted.
minimum_validity	0.15	Minimum validity for keeping a tie point, expressed as proportion of matching pixels in the reference water body edge.
minimum_importance	3	Minimum importance for keeping a tie point, obtained from importance evaluation tests.

**Table A5:** Parameters used for fitting transformation parameters.

Parameter	Value	Explanation
minimum_num_tp	2	Minimum number of tie points to correct an image; increase if additional transformation parameters are searched.
rotation_span	1.5	Bound for rotation parameter value to be searched for.
eucl_dist_threshold_small	3	Maximum residual error for a tie point.
percentile_threshold	80	Frequency of high residual error in all combinations of tie points, below which tie points are rejected.

## References

[1] Müller R., Krauß T., Schneider M., Reinartz P. (2012). Automated georeferencing of optical satellite data with integrated sensor model improvement. Photogramm. Eng. Remote Sens..

[2] Smyth M., Logan T.L. (2020). ECOSTRESS Science Meeting L1B Geolocation Review. https://ecostress.jpl.nasa.gov/downloads/science_team_meetings/2020/fall_pres/day1/05_Ecostress_L1B_Geolocation_01DEC2020.pdf.

[3] Leprince S., Barbot S., Ayoub F., Avouac J.P. (2007). Automatic and precise orthorectification, coregistration, and subpixel correlation of satellite images, application to ground deformation measurements. IEEE Trans. Geosci. Remote Sens..

[4] Logan T., Smyth M. L1 Calibration and Geolocation Review. Proceedings of the ECOSTRESS Science Meeting November.

[5] Long T., Jiao W., He G., Zhang Z. (2016). A fast and reliable matching method for automated georeferencing of remotely-sensed imagery. Remote Sens..

[6] Heipke C. (1997). Automation of interior, relative, and absolute orientation. ISPRS J. Photogramm. Remote Sens..

[7] Dowman I. (1998). Automating image registration and absolute orientation: Solutions and problems. Photogramm. Rec..

[8] Turner D., Lucieer A., Malenovskỳ Z., King D.H., Robinson S.A. (2014). Spatial co-registration of ultra-high resolution visible, multispectral and thermal images acquired with a micro-UAV over Antarctic moss beds. Remote Sens..

[9] Torgersen C.E., Faux R.N., McIntosh B.A., Poage N.J., Norton D.J. (2001). Airborne thermal remote sensing for water temperature assessment in rivers and streams. Remote Sens. Environ..

[10] Tsanakas J.A., Ha L.D., Al Shakarchi F. (2017). Advanced inspection of photovoltaic installations by aerial triangulation and terrestrial georeferencing of thermal/visual imagery. Renew. Energy.

[11] Wang K., Jiang Q.G., Yu D.H., Yang Q.L., Wang L., Han T.C., Xu X.Y. (2019). Detecting daytime and nighttime land surface temperature anomalies using thermal infrared remote sensing in Dandong geothermal prospect. Int. J. Appl. Earth Obs. Geoinf..

[12] Coolbaugh M., Kratt C., Fallacaro A., Calvin W., Taranik J. (2007). Detection of geothermal anomalies using Advanced Spaceborne Thermal Emission and Reflection Radiometer (ASTER) thermal infrared images at Bradys Hot Springs, Nevada, USA. Remote Sens. Environ..

[13] Crippen R.E., Hook S.J., Fielding E.J. (2007). Nighttime ASTER thermal imagery as an elevation surrogate for filling SRTM DEM voids. Geophys. Res. Lett..

[14] Shi J., Hu C. (2021). Evaluation of ECOSTRESS thermal data over South Florida estuaries. Sensors.

[15] Dugdale S.J. (2016). A practitioner’s guide to thermal infrared remote sensing of rivers and streams: Recent advances, precautions and considerations. Wiley Interdiscip. Rev..

[16] Dugdale S.J., Bergeron N.E., St-Hilaire A. (2015). Spatial distribution of thermal refuges analysed in relation to riverscape hydromorphology using airborne thermal infrared imagery. Remote Sens. Environ..

[17] Zhao Q., Wentz E.A. (2016). A MODIS/ASTER Airborne Simulator (MASTER) Imagery for Urban Heat Island Research. Data.

[18] Khanal S., Fulton J., Shearer S. (2017). An overview of current and potential applications of thermal remote sensing in precision agriculture. Comput. Electron. Agric..

[19] Burt J.E., White J., Allord G., Then K.M., Zhu A.X. (2020). Automated and semi-automated map georeferencing. Cartogr. Geogr. Inf. Sci..

[20] Kuenzer C., Dech S. (2013). Theoretical Background of Thermal Infrared Remote Sensing. Thermal Infrared Remote Sensing: Sensors, Methods, Applications.

[21] Engineering ToolBox (2003). Water—Thermophysical Properties. https://www.engineeringtoolbox.com/water-thermal-properties-d_162.html.

[22] Government of Kenya & UNDP (2021). Rising Water Levels in Kenya’s Rift Valley Lakes, Turkwel Gorge dam and Lake Victoria. https://ir-library.ku.ac.ke/handle/123456789/22851.

[23] Oliver J.A., Pivot F.C., Tan Q., Cantin A.S., Wooster M.J., Johnston J.M. (2022). A Machine Learning Approach to Waterbody Segmentation in Thermal Infrared Imagery in Support of Tactical Wildfire Mapping. Remote Sens..

[24] NASA/METI/AIST/Japan Spacesystems and U.S./Japan ASTER Science Team (2019). ASTER Global Water Bodies Database V001. https://cmr.earthdata.nasa.gov/search/concepts/C1575734433-LPDAAC_ECS.html.

[25] OpenCV Canny Edge, n.d. https://docs.opencv.org/3.4/da/d22/tutorial_py_canny.html.

[26] Hulley G., Freepartner R. (2022). ECOsystem Spaceborne Thermal Radiometer Experiment on Space Station (ECOSTRESS) Mission Level 2 Product User Guide.

[27] Hulley G.C., Göttsche F.M., Rivera G., Hook S.J., Freepartner R.J., Martin M.A., Cawse-Nicholson K., Johnson W.R. (2021). Validation and quality assessment of the ECOSTRESS level-2 land surface temperature and emissivity product. IEEE Trans. Geosci. Remote Sens..

[28] Drusch M., Del Bello U., Carlier S., Colin O., Fernandez V., Gascon F., Hoersch B., Isola C., Laberinti P., Martimort P. (2012). Sentinel-2: ESA’s Optical High-Resolution Mission for GMES Operational Services. Remote Sens. Environ..

[29] Berger M., Moreno J., Johannessen J.A., Levelt P.F., Hanssen R.F. (2012). ESA’s Sentinel missions in support of Earth system science. Remote Sens. Environ..

[30] S2 MSI ESL Team Data Quality Report Sentinel-2 L1C MSI. July 2022. https://sentinels.copernicus.eu/documents/247904/4766914/OMPC.CS.DQR.001.03-2022+-+i74r0+-+MSI+L1C+DQR+April+2022.pdf.

[31] Gorelick N., Hancher M., Dixon M., Ilyushchenko S., Thau D., Moore R. (2017). Google Earth Engine: Planetary-scale geospatial analysis for everyone. Remote Sens. Environ..

[32] ESA Sentinel-2 MSI Level-2A Algorithm Overview. https://sentinel.esa.int/web/sentinel/technical-guides/sentinel-2-msi/level-2a/algorithm..

[33] Louis J., Charantonis A., Berthelot B. Cloud Detection for Sentinel-2. Proceedings of the ESA Living Planet Symposium.

